# Microbial diversity, antimicrobial resistance and zoonotic implications of the reptile gut microbiota: an updated review

**DOI:** 10.3389/fmicb.2026.1913228

**Published:** 2026-09-01

**Authors:** Dexu Kong, Yanhui Zhang, Zheng Li, Lingling Chen, Jiaqi Nie, Xiao Jiang, Henan Cao, Yao Ma

**Affiliations:** 1 School of Basic Medicine, Qingdao University, Qingdao, Shandong, China; 2 Qingdao Women and Children’s Hospital, Qingdao University, Qingdao, China; 3 Institute of Microbiology, Chinese Academy of Sciences, Beijing, China; 4 School of Basic Medical Sciences, Tianjin Medical University, Tianjin, China

**Keywords:** antimicrobial resistance, companion animal, gut microbiota, reptile, zoonosis

## Abstract

The reptilian gastrointestinal tract harbors a complex and dynamic ecosystem of microorganisms that plays a fundamental and multifaceted role in host nutrition, immune function, and overall physiological homeostasis. This gut microbiome exhibited remarkable phylogenetic and functional diversity, intricately shaped by a confluence of host evolutionary history, dietary strategy, environmental context, captive status, and life history traits. Beyond its critical importance for reptilian health and fitness, this internal microbial reservoir is of significant and growing concern from a public health perspective, serving as a major source of zoonotic pathogens, most notably non-typhoidal Salmonella, and as a critical and underexplored hotspot for the emergence, amplification, and dissemination of antimicrobial resistance genes (ARGs), a dimension that forms the central theme of this review and is systematically examined across host ecology, captive management, and the global pet trade continuum. This comprehensive review synthesizes contemporary research on the gut microbiota across key reptilian taxa, including popular companion species such as lizards such as *Eublepharis macularius* and *Tiliqua scincoides*, snakes such as *Pantherophis guttatus*, *Python regius* and *chelonians*. We undertake a detailed analysis of the foundational drivers shaping microbial community structure, assembly and stability, exploring the delicate balance between core symbiotic residents, putatively beneficial probiotic candidates, and pathogenic entities. A critical and extensive focus is placed on the distribution, ecological drivers, and transmission pathways of antimicrobial resistance genes within this ecosystem, highlighting its underappreciated role in the global One Health continuum. The review further elaborates on the indispensable metabolic contributions of the microbiota to host fitness, the complex tripartite interactions involving host, microbiome and parasitic helminths or protozoa, and the implications of dysbiosis. Finally, we evaluated practical and emerging strategies for targeted microbiome modulation aimed at enhancing captive management, supporting conservation breeding outcomes, mitigating zoonotic risks and promoting sustainable herpetoculture.

## 1 Introduction

Reptiles comprise more than 11,000 extant species. They occupy terrestrial, aquatic, arboreal and fossorial niches across all continents except Antarctica. This remarkable adaptive radiation has been accompanied by diverse dietary strategies, digestive physiologies and life history traits (Vitt and Pianka, 2005; Colston and Jackson, 2016; Roll et al., 2017). The gastrointestinal tract of reptiles harbors complex microbial communities, including bacteria, archaea, fungi and viruses. These microorganisms together with the host form a holobiont, and their collective functions extend beyond those encoded by the host genome alone (Colston and Jackson, 2016; Eliades et al., 2021; Dubin and Pamer, 2014). Recent advances in hologenome theory emphasize that the host and its symbiotic microorganisms constitute a single evolutionary unit. The gut microbiota plays an integral role in host nutrition, immune development and metabolic adaptation. Reptiles exhibit distinctive physiological traits, such as ectothermy, intermittent feeding and remarkable fasting tolerance. These traits may shape their gut microbial communities in ways that require dedicated research frameworks rather than simple extrapolation from endothermic vertebrates (Secor, 2005).

Both host phylogeny and dietary strategy influence gut microbial community composition in reptiles. Disentangling their relative contributions remains challenging because diet and evolutionary history are often confounded across lineages (Roll et al., 2017; Kohl et al., 2017). Phylogenetically controlled comparisons within closely related taxa are therefore essential. For example, the gut microbiota of herbivorous lizards differs markedly from that of closely related insectivorous or omnivorous species, as demonstrated in a controlled study by Kohl et al. (2017). In captivity, the reptile gut microbiome is further shaped by artificial diets, reduced environmental microbial exposure and husbandry practices. Studies on captive lizards have documented significant shifts in microbial diversity and composition compared to wild conspecifics (Hernández et al., 2024; Zhang et al., 2019). In snakes, most existing data come from captive or commercially farmed individuals. Wild snake populations remain severely understudied, although pioneering work has revealed taxon specific and geographic variation in gut microbial communities (Smith et al., 2021; Colston et al., 2025). A recent synthesis calls for expanded field surveys of wild snake microbiomes to fill this knowledge gap (Colston et al., 2025; Pees et al., 2023).

From a public health perspective, reptiles are well established asymptomatic carriers of zoonotic enteric pathogens, most notably non typhoidal *Salmonella enterica* (Pees et al., 2023; Marshall et al., 2020). The global trade in live reptiles, which involves millions of animals annually for the pet industry, creates extensive interfaces between humans and captive reptiles (Corrente et al., 2017). This interface has been repeatedly linked to reptile associated salmonellosis in vulnerable populations, including young children and immunocompromised individuals (Mermin et al., 2004; Casalino et al., 2021).

Beyond well recognized zoonotic pathogens, the reptilian gut also serves as an underexplored reservoir for antimicrobial resistance genes (ARGs) and multidrug resistant bacteria. Studies have isolated extended spectrum beta lactamase producing *Escherichia coli* and *Salmonella* from pet reptiles, as well as from farmed turtles and lizards (Pees et al., 2023; Dróżdż et al., 2021; Choi et al., 2023). The reptilian gut environment presents conditions that may favor horizontal gene transfer. Intermittent feeding leads to prolonged fasting periods, followed by rapid nutrient influx. Ectothermy causes gut temperature to fluctuate with ambient conditions. These factors can affect plasmid conjugation efficiency and the stability of mobile genetic elements, potentially creating a unique selective landscape for ARG dissemination. Compared to the well-studied livestock and human gut resistomes, the reptilian gut resistome remains largely uncharacterized. Its composition, ecological drivers and transmission pathways along the pet trade chain represent a critical gap in the One Health surveillance framework. This gap is particularly concerning given that recent large-scale surveys across diverse vertebrate hosts have revealed that host ecology, geography, and anthropogenic activities profoundly shape the composition and dissemination dynamics of gut resistomes and mobile genetic elements (MGEs) (Yang et al., 2022; Yang et al., 2024). For instance, comprehensive metagenomic analyses of mammalian gut microbiomes across altitudinal gradients on the Tibetan Plateau have demonstrated that environmental stressors and host adaptation significantly influence both microbial community structure and ARG carriage patterns (Tian et al., 2025a), while population-level studies in humans have shown that industrialization and socioeconomic factors drive not only taxonomic shifts but also the expansion of MGE-associated resistance genes within the gut ecosystem (Tian et al., 2025b; Tian and Zhang, 2025). These findings underscore the need to situate reptilian gut resistome research within broader comparative frameworks that account for ecological context, anthropogenic pressure, and the evolutionary dynamics of MGE-mediated gene flow. Accordingly, the present review adopts antimicrobial resistance and One Health considerations not as a peripheral topic but as a unifying analytical lens through which the ecological and functional dimensions of the reptilian gut microbiome are interpreted, ensuring that the AMR framing stated in our scope is consistently engaged throughout the text.

This review synthesizes the current state of knowledge regarding the composition, assembly and functional significance of the reptilian gut microbiome, with a particular emphasis on microbial diversity, antimicrobial resistance and zoonotic potential. Throughout this review, we use ‘gut microbiota’ to refer to the microbial community itself and ‘gut microbiome’ to encompass the collective genetic material and functional potential of these microorganisms, in line with current ecological nomenclature. We first outline the ecological and evolutionary factors that structure these microbial communities, including host phylogeny, diet, environmental context, captive status and life history stage. We then examine the functional ecology of the gut microbiota, addressing its metabolic contributions, the interplay among host, microbiome and parasites, and the potential of the reptilian gut as a source of novel probiotic candidates. A dedicated section is devoted to antimicrobial resistance in the reptilian gut, tracing the sources, reservoirs and transmission pathways of resistance genes and their public health relevance. Throughout, we highlight knowledge gaps and methodological challenges, and we propose future research priorities for translating microbiome science into evidence based strategies for captive management, conservation breeding and zoonotic risk mitigation.

## 2 Factors shaping the reptilian gut microbiome

### 2.1 Host phylogeny and gut anatomy

The architecture and population dynamics of the reptilian gut microbiome are not stochastic assemblages but are shaped by a series of deterministic filters acting at spatial, temporal, and biological scales (Chen et al., 2022). At the broadest macroevolutionary level, host phylogeny imposes a deep, historical constraint, with discernible phylum-level patterns often differentiating major orders such as Squamata (lizards and snakes) and Testudines (turtles and tortoises). This phylogenetic signal is intrinsically linked to and manifested through diverging gastrointestinal tract anatomies. The gut of a carnivorous snake such as corn snake and kingsnake, is relatively short, linear, and enzymatically focused, presenting fundamentally different physicochemical niches (transit time, pH, bile acids) compared to the elongated, voluminous hindgut of a herbivorous tortoise, which is specialized for microbial fermentation (Tang et al., 2019). These physiological differences are directly reflected in gut microbial communities. Herbivorous reptiles typically host richer, more diverse, and functionally complex consortia dominated by fermentative taxa, whereas carnivorous species harbor less diverse communities specialized in proteolysis and lipid metabolism (Costello et al., 2010). Compared with mammals, reptiles exhibit several distinctive features. The strong dependence of microbial metabolic rates on ambient temperature in ectotherms contrasts with the relatively constant-temperature environment of the mammalian gut, potentially affecting community dynamics and functional outputs. Furthermore, the extraordinary fasting tolerance of many reptiles, far exceeding that of similarly sized mammals or birds, imposes feast-and-famine cycles on gut microbes that have few parallels in endothermic vertebrates. These comparative distinctions remain poorly characterized at the molecular and functional levels and represent an important direction for future research.

Epidemiological meta-analyses starkly underscore these deep ecological differences, revealing significant and consistent variation in the carriage of the enteric pathogen *Salmonella*. A comprehensive review of over 12,000 reptiles found prevalence was highest in snakes (56.0%), intermediate in lizards (36.9%) and tortoises (34.2%), and substantially lower in aquatic turtles (18.6%) and crocodilians (9.0%) (Pees et al., 2023). This gradient likely arises from a complex interplay of dietary habit, gut transit time, host immune system architecture, and differential environmental exposure risk, though the precise mechanistic contributions of each factor remain an active area of investigation.

Beyond prevalence patterns of zoonotic pathogens, host phylogenetic gradients also have direct implications for antimicrobial resistance gene carriage and dissemination. The differential gut transit times, luminal pH, and microbial community densities across carnivorous, omnivorous, and herbivorous reptiles create distinct physicochemical environments that may influence the efficiency of horizontal gene transfer and the stability of mobile genetic elements carrying resistance determinants. For instance, the prolonged retention times and dense microbial biomass characteristic of herbivorous hindgut fermenters may facilitate more frequent plasmid-mediated conjugation events compared to the short-transit, enzyme-dominated guts of strict carnivores. This phylogenetic and physiological linkage between host anatomy and resistance ecology remains virtually unexplored but represents a critical gap in One Health surveillance, given that the international pet trade moves phylogenetically diverse reptile species across geographical boundaries, each carrying a unique resistome signature shaped by its evolutionary heritage.

### 2.2 Captivity as a modulator

Dominant anthropogenic force restructuring the microbiome is the transition from a free-ranging wild existence to a captive environment. Captive reptiles consistently exhibit altered, and frequently depauperate, microbial communities relative to their free-ranging conspecifics, a phenomenon termed the “captivity effect” (Dallas and Warne, 2023). This shift is attributed to multiple anthropogenic factors: nutritionally and microbiologically simplified diets; artificial enclosures that restrict environmental microbial exchange; routine prophylactic antimicrobial use; and chronic captivity-induced stress, which collectively alter gut permeability, motility, and immune surveillance (Puvača, 2022). Notably, the prevalence of *Salmonella* and other enteric pathogens is frequently reported to be significantly elevated in captive populations (Baling and Mitchell, 2021). This phenomenon may be driven by factors such as increased host density facilitating efficient fecal-oral transmission, the common practice of feeding frozen–thawed rodents or other prey items that can be contaminated during production, and stress-induced shedding dynamics that enhance bacterial excretion from subclinical carriers, thereby increasing environmental load and transmission risk within closed systems (Sodagari et al., 2020). Critically, the captive environment also serves as a selective arena for antimicrobial resistance. Prophylactic or therapeutic antibiotic use, which remains common in some commercial reptile breeding facilities despite growing stewardship awareness, imposes direct selective pressure on gut microbial communities. This pressure not only enriches for resistant commensals such as *E. coli* and *Enterococcus* spp. but also promotes the co-selection of resistance genes linked on mobile genetic elements. Moreover, the closed and densely populated nature of captive colonies facilitates rapid dissemination of resistant strains once they emerge, creating a reservoir effect whereby the captive gut resistome may become more abundant and diverse than that of wild conspecifics. This captivity-AMR nexus has direct implications for the pet trade pathway, as animals leaving breeding facilities may carry enriched resistance gene pools into household environments, thereby establishing a direct link between captive management practices and public health exposure risk.

### 2.3 Dietary niche and microbial functional guilds

Diet acts as the most powerful proximate selector, directly enriching for microbial taxa equipped with the enzymatic machinery to metabolize the most abundant available substrates (Youngblut et al., 2021). Herbivorous reptiles, such as the blue-tongued skink (*Tiliqua scincoides*) or the omnivorous but plant-heavy diets of crested geckos (*Correlophus ciliatus*), harbored microbiota enriched with members of the phyla Firmicutes (notably cellulolytic and hemicellulolytic *Clostridia* clusters) and Bacteroidetes, which encoded extensive repertoires of carbohydrate-active enzymes (CAZymes) for the degradation of complex plant structural polysaccharides like cellulose, hemicellulose and pectin (Costello et al., 2010; Moeller et al., 2021). In stark contrast, the guts of strict carnivorous snakes like ball pythons (*Python regius*) or hognose snakes (*Heterodon nasicus*) are typically dominated by Proteobacteria and Fusobacteria, taxa specialized in the efficient breakdown of proteins, peptides and lipids, with a correspondingly reduced capacity for carbohydrate metabolism (Cong et al., 2025). Omnivorous species, including many box turtles, monitors, and some skinks, display hybrid or flexible community structures capable of adapting to nutritionally mixed inputs. Through specific formulations such as pelleted feeds for herbivores, insect-based gels for frugivorous geckos, or single-source rodent diets for snakes, commercial diets further shaped these microbial communities. This usually resulted in profiles that markedly diverge from wild-type microbiota and may, over time, lead to suboptimal nutrient extraction or dysbiosis (Puvača, 2022). Dietary niche also modulates the trajectory of antimicrobial resistance gene acquisition. Omnivorous and herbivorous reptiles, with their higher microbial diversity and fermentative communities, harbor larger and more varied mobilomes, the collection of mobile genetic elements within a microbiome, compared to carnivorous species with simpler communities. This increased genetic exchange potential within complex herbivore guts may facilitate the capture and retention of ARGs from environmental or dietary sources, including commercially produced plant-based feeds that may contain sub-inhibitory concentrations of agricultural antibiotics or resistant bacterial contaminants. Thus, diet not only shapes microbial community composition but also influences the quantitative and qualitative landscape of the gut resistome, with herbivorous and omnivorous species potentially serving as more efficient ARG reservoirs than strict carnivores, a hypothesis that warrants systematic testing across dietary guilds.

### 2.4 Ontogeny and seasonal cycles

Microbial succession and temporal dynamics are inherent properties of the gut ecosystem, varying predictably with ontogenetic stage and cyclical physiological states (Hernández-Gómez et al., 2017). Neonates and hatchlings acquire their pioneer microbiota through a combination of vertical transmission and horizontal acquisition from the nest environment and initial food items (Bosch et al., 2016). This initial, stochastic community undergoes progressive maturation, stabilization, and selection as the juvenile grows, its diet diversifies from yolk reserves to solid food, and its adaptive immune system develops tolerance to commensal antigens (López-Rojas et al., 2025). Furthermore, profound physiological cycles impose rhythmic changes on the microbiome. Hibernation in temperate species like the milk snake (*Lampropeltis triangulum*) or gopher snake (*Pituophis melanoleucus*) is associated with a dramatic reduction in microbial biomass, diversity, and a distinct shift in composition, mirroring the host’s extended fasted state, depressed metabolism, and lower gut motility (Wollenberg Valero et al., 2019). Similarly, the elevated energetic and nutritional demands of vitellogenesis and egg production in females can selectively enrich for microbial communities with enhanced nutrient assimilation capabilities, particularly for lipids and calcium, demonstrating a functional link between reproductive physiology and microbiome state (Wolf et al., 2014).

### 2.5 The concept of a core microbiome in reptiles

Despite the considerable interspecific and intraspecific variation, certain bacterial phyla, primarily Firmicutes, Bacteroidetes, Proteobacteria and Actinobacteria, consistently emerge as dominant across the reptilian lineage (Amato et al., 2015). However, the concept of a universal, taxonomically defined “core microbiome” at fine phylogenetic levels (genus or species) is elusive due to the immense ecological and phylogenetic diversity of reptiles (Bletz et al., 2016). A more robust and functionally meaningful concept may be the ecological core or functional core, whereby phylogenetically distinct microbial groups converge to perform host essential functions in animals sharing similar dietary strategies or gut morphologies. These functions include fiber fermentation, vitamin synthesis, bile acid metabolism, and immune priming. Although this concept of convergent community function was initially developed from mammalian studies, it has been speculated to extend to reptiles (Muegge et al., 2011). Direct experimental validation in the reptilian gut remains largely absent and represents an important area for future investigation. Within more closely related groups, such as congeneric snake species or geographically proximate populations of the same herbivorous tortoise species, shared evolutionary history and ecology can promote the conservation of specific microbial lineages, suggesting a role for host genetics in shaping microbial compatibility (Bosch et al., 2016).

### 2.6 Community sequencing and isolate-based evidence

#### 2.6.1 Dietary niche as the evolutionary driver of community structure

The Animal Microbiome Database^1^ is a database covering bacterial 16S rRNA gene profiles from various animal species to assess the relationship between the gut microbiota and animal hosts, which contains 10,478 bacterial taxa from 467 animal species. Analysis of 16S rRNA from AMDB establishes host dietary niche as the principal, overarching determinant of reptilian gut microbiome composition (Table 1; Supplementary Table S1). Comparative studies have shown that there are significant differences in the composition of gut microbiota among carnivorous, herbivorous, and omnivorous reptiles. Carnivorous animals such as Burmese pythons and vipers possess gut microbiota rich in taxa specialized in protein and fat catabolism, such as Fusobacteria. In contrast, herbivores such as Aldabra giant tortoise and gopher tortoise are dominated by fermentative Firmicutes and Bacteroidetes. Omnivorous species like the bearded dragon lizard exhibit intermediate and mixed characteristics (Table 1; Supplementary Table S1). This diet-microbiome axis represented a core evolutionary adaptation, where microbial symbionts expanded the metabolic capabilities of the host.

**Table 1:** Summary of publicly available 16S rRNA gene datasets from reptile gut samples analyzed in this review.

Host species	Family	Diet	Status	BioProject	Microbiome features	Ref
*Aldabrachelys gigantea* (Aldabra giant tortoise)	Testudinidae	Herbivore	Captive	PRJNA362214 (*n* = 6)	Firmicutes, Bacteroidetes dominated; high fermentative potential	Yang et al. (2022)
*Basiliscus plumifrons* (Green basilisk)	Iguanidae	Omnivore	Captive	PRJEB35449 (*n* = 1)	Intermediate community; mixed dietary signatures	Yang et al. (2022)
*Crocodylus moreletii* (Morelet’s crocodile)	Crocodylidae	Carnivore	Captive	PRJEB29403 (*n* = 1)	Proteobacteria enriched; low fermentative capacity	Yang et al. (2022)
*Crocodylus niloticus* (Nile crocodile)	Crocodylidae	Carnivore	Wild	PRJEB29403 (*n* = 1)	Wild-type community; distinct from captive crocodilians	Yang et al. (2022)
*Crocodylus rhombifer* (Cuban crocodile)	Crocodylidae	Carnivore	Captive	PRJEB35449 (*n* = 4)	Captivity-associated shifts; reduced diversity	Yang et al. (2022)
*Cuora bourreti* (Indochinese box turtle)	Geoemydidae	Omnivore	Captive	PRJEB35449 (*n* = 1)	Mixed dietary signals; omnivorous profile	Yang et al. (2022)
*Cyclura collei* (Jamaican iguana)	Iguanidae	Herbivore	Captive	PRJEB35449 (*n* = 1)	Fermentative Firmicutes enriched	Yang et al. (2022)
*Deinagkistrodon acutus* (Chinese moccasin)	Viperidae	Carnivore	Captive	PRJNA516815 (*n* = 5)	Fusobacteria, Proteobacteria dominated	Zhang et al. (2019)
*Drymarchon corais* (Indigo snake)	Colubridae	Carnivore	Captive	PRJEB35449 (*n* = 1)	Carnivore-typical; low carbohydrate metabolism	Yang et al. (2022)
*Elaphe carinata* (King ratsnake)	Colubridae	Carnivore	Captive	PRJNA516815 (*n* = 6)	Proteobacteria, Fusobacteria; captivity-associated	Zhang et al. (2019)
*Eublepharis macularius* (Leopard gecko)	Eublepharidae	Carnivore	Captive	PRJEB35449 (*n* = 10)	High inter-individual variation; captivity effects documented	Hernández et al. (2024); Dallas and Warne (2023)
*Heloderma charlesbogerti* (Guatemalan beaded lizard)	Helodermatidae	Carnivore	Captive	PRJEB35449 (*n* = 3)	Carnivore-associated taxa; limited diversity	Yang et al. (2022)
*Hemidactylus frenatus* (Common house gecko)	Gekkonidae	Carnivore	Wild	PRJEB29403 (*n* = 1)	Wild community reference; distinct from captive geckos	Yang et al. (2022)
*Lampropeltis getula* (King snake)	Colubridae	Carnivore	Captive	PRJEB35449 (*n* = 1)	Carnivore profile; captivity influences	Yang et al. (2022)
*Manouria impressa* (Impressed tortoise)	Testudinidae	Herbivore	Captive	PRJEB35449 (*n* = 2)	Bacteroidetes, Firmicutes enriched; fiber fermenters	Yang et al. (2022)
*Morelia viridis* (Green tree python)	Pythonidae	Carnivore	Captive	PRJEB35449 (*n* = 1)	Proteobacteria, Fusobacteria typical of pythons	Yang et al. (2022)
*Naja atra* (Chinese cobra)	Elapidae	Carnivore	Captive	PRJNA516815 (*n* = 4)	Proteobacteria dominated; low metabolic diversity	Zhang et al. (2019)
*Nerodia sipedon* (Northern watersnake)	Colubridae	Carnivore	Captive	PRJEB35449 (*n* = 1)	Aquatic influence; Proteobacteria prominent	Yang et al. (2022)
*Physignathus coccincus* (Chinese water dragon)	Agamidae	Carnivore	Captive	PRJEB35449 (*n* = 1)	Insectivorous/carnivorous profile	Yang et al. (2022)
*Podarcis siculus* (Italian wall lizard)	Lacertidae	Carnivore	Wild	PRJEB29403 (*n* = 1)	Wild-type community; strong diet-correlated shifts	Kohl et al. (2017)
*Ptyas mucosa* (Oriental ratsnake)	Colubridae	Carnivore	Captive	PRJNA516815 (*n* = 4)	Carnivore-typical; Proteobacteria dominated	Zhang et al. (2019)
*Rhacodactylus leachianus* (New Caledonian giant gecko)	Diplodactylidae	Omnivore	Captive	PRJEB35449 (*n* = 1)	Mixed community reflecting omnivorous diet	Yang et al. (2022)
*Shinisaurus crocodilurus* (Chinese crocodile lizard)	Shinisauridae	Carnivore	Captive	PRJEB35449 (*n* = 2)	Captive-associated profile; limited data	Yang et al. (2022)
*Stigmochelys pardalis* (Leopard tortoise)	Testudinidae	Herbivore	Captive	PRJEB35449 (*n* = 1)	Firmicutes, Bacteroidetes; fermentative community	Yang et al. (2022)
*Testudo hermanni* (Hermann’s tortoise)	Testudinidae	Omnivore	Wild	PRJEB29403 (*n* = 1)	Wild omnivore reference; diverse community	Yang et al. (2022)
*Trioceros melleri* (Meller’s chameleon)	Chameleonidae	Carnivore	Captive	PRJEB35449 (*n* = 7)	Insectivore-associated; captivity effects documented	Yang et al. (2022)
*Uroplatus lineatus* (Lined flat-tail gecko)	Gekkonidae	Carnivore	Captive	PRJEB35449 (*n* = 2)	Captive gecko profile; limited diversity	Yang et al. (2022)
*Vipera ursinii* (Meadow viper)	Viperidae	Carnivore	Wild	PRJEB29403 (*n* = 2)	Distinct from captive snakes; higher Proteobacteria diversity	Smith et al. (2021); Colston et al. (2025)
*Zamenis longissimus* (Aesculapian snake)	Colubridae	Carnivore	Wild	PRJEB29403 (*n* = 1)	Wild snake reference; limited data	Yang et al. (2022)

A complete list of all individual sample accession numbers (SRS and ERS identifiers) with corresponding metadata (sample ID, individual specimen details, and sequencing run information) is provided in Supplementary Table S1.

#### 2.6.2 The captive environment as a potent anthropogenic modulator

The state of captivity emerged as a major extrinsic factor reshaping the gut ecosystem. A significant portion of sequence data originates from animals in managed care (Table 1; Supplementary Table S1). This environment has brought about profound changes, with standardized feed replacing diversified feed, environmental microbial exposure becoming homogenized, and stress factors associated with confinement affecting the physiological functions of the host. Consequently, captive microbiomes often exhibit reduced alpha diversity and taxonomic shifts, poorly representing wild conspecifics. This anthropogenic influence has direct implications for pathogen dynamics and zoonotic risk, as captivity can inadvertently alter the abundance of bacterial groups, including potential pathogens.

#### 2.6.3 Integrative insights from community sequencing and isolate collections

The combination of 16S rRNA community surveys and culture-based isolate collections provides complementary perspectives on the reptilian gut ecosystem. Community sequencing, as summarized in Table 1, reveals dietary niche and captivity as dominant forces shaping taxonomic composition. Bacterial isolates cultured from reptiles, including *S. enterica* serovars, *E. coli*, *Klebsiella pneumoniae*, *Pseudomonas aeruginosa*, and *Acinetobacter baumannii*, confirm that reptiles carry both established zoonotic pathogens and Gram-negative bacteria known to acquire antimicrobial resistance genes. These isolate-based findings provide a direct link to the AMR crisis and underscore the relevance of the reptilian gut to One Health surveillance efforts.

## 3 Functional ecology of the reptilian gut microbiome

### 3.1 Probiotic candidates from the reptilian gut

**Figure 1:**
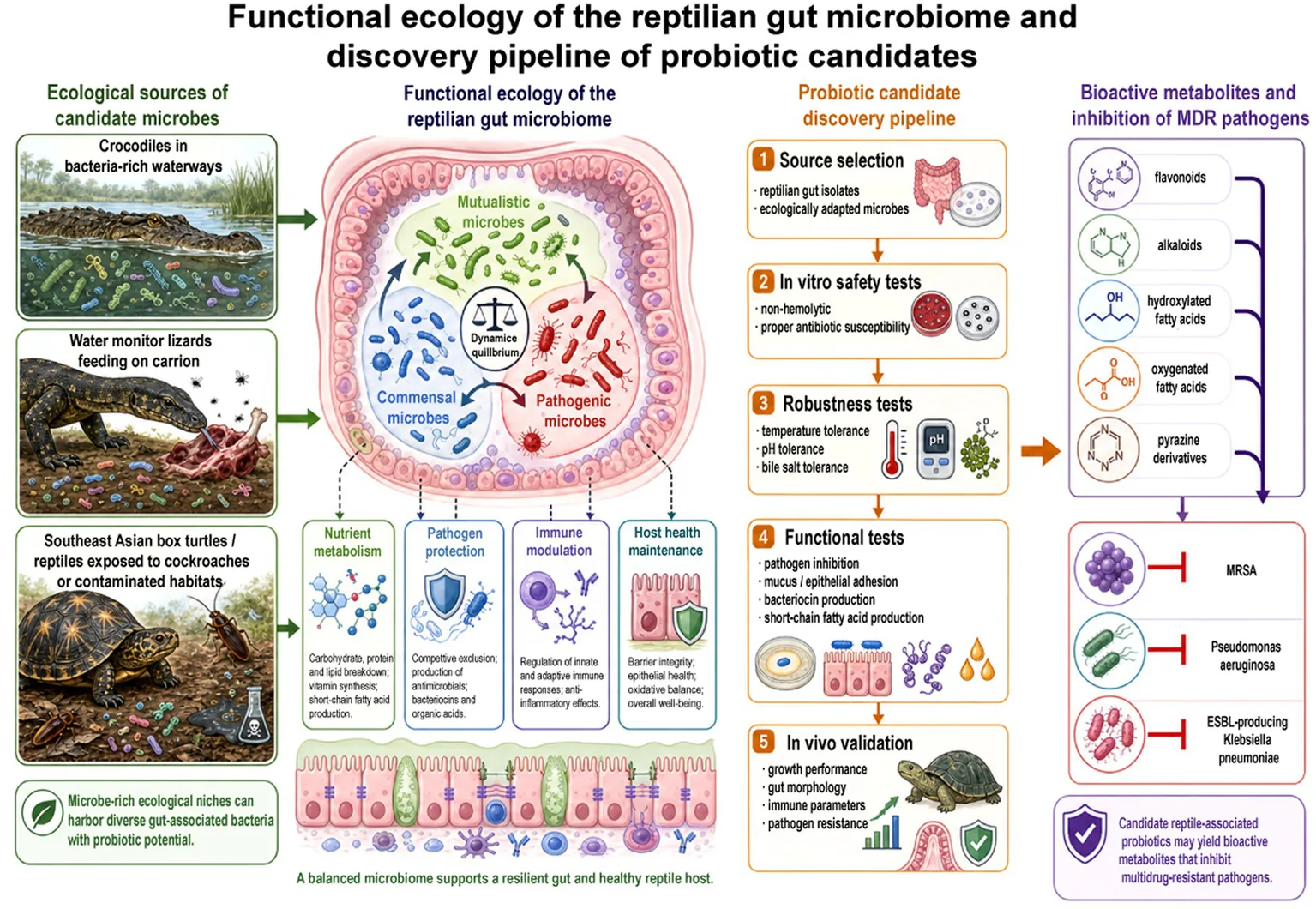
Schematic workflow for sourcing and characterizing candidate probiotics from the reptilian gut microbiome. The gut microbiota and the host immune system are in a dynamic, bidirectional regulatory relationship: The microbiota modulates immune development and function, while the host immune system shapes microbial community composition. Candidate probiotics sourced from reptiles inhabiting microbe-rich or extreme environments are screened through sequential *in vitro* assessments of safety, functional activity, and gut-relevant stress tolerance, followed by *in vivo* validation in target reptile species.

Animals are exposed to a vast array of microorganisms in their natural environment, and their antimicrobial defense capabilities may stem from either (i) the host immune system and/or (ii) the gut microbiota. The gut microbiota is crucial for host functions, offering multiple benefits including nutrient metabolism, pathogen protection, immune modulation, and potential protection against various health issues. Thus, animals living in environments rich in microorganisms such as cockroaches, scavengers such as water monitor lizards, or environments contaminated with heavy metals and consuming carrion such as crocodiles may have gut bacteria that serve as a unique source of novel bioactive molecules. These molecules could potentially exhibit significant efficacy against multidrug-resistant bacteria, which often lead to high mortality rates, as illustrated in Figure 1. Hence, this field represents an important research direction worthy of further exploration.

The reptilian gut microbiome constituted a theater of constant, multifaceted interaction, hosting a delicate and dynamic balance between mutualistic, commensal, and pathogenic entities. A promising and growing avenue of research focused on the systematic identification of endogenous probiotic candidates. This hypothesis-driven approach is grounded in the perspective of ecological evolution theory, suggesting that reptile species that thrive in environments challenging to microorganisms, such as crocodiles inhabiting bacteria-filled waterways, water monitor lizards (*Varanus salvator*) that feed on decaying corpses, or species that prey on cockroaches, are likely to have co-evolved with microorganisms, or their microbial communities have evolved to produce defensive compounds in response to persistent microbial challenges. Substantiating this, compelling studies have isolated cultivable gut bacteria from the water monitor lizard and the Southeast Asian box turtle (*Cuora amboinensis*) whose cell-free culture metabolites exhibit potent, broad-spectrum antibacterial activity *in vitro* against critical human pathogens like methicillin-resistant *Staphylococcus aureus* (MRSA), *Pseudomonas aeruginosa*, and extended-spectrum beta-lactamase (ESBL)-producing *Klebsiella pneumoniae* (Akbar et al., 2019a; Akbar et al., 2019b). Metabolomic profiling of these active supernatants had identified a suite of bioactive molecules, including flavonoids, alkaloids, specific hydroxylated and oxygenated fatty acids, and pyrazine derivatives, some of which had known antimicrobial properties. The systematic screening of probiotics derived from reptiles involves a sequential process. First, target sources are selected from hosts that exhibit stress resistance or inhabit specific ecological niches. Next, rigorous *in vitro* assessments are conducted to evaluate safety parameters, including the absence of hemolysis and appropriate antibiotic susceptibility. Robustness is assessed through tolerance to reptile gut-relevant conditions such as temperature, pH, and bile salt concentrations. Functionality is determined by quantitative pathogen inhibition tests, adhesion to mucus or intestinal epithelial cell lines, and the production of antimicrobial compounds like bacteriocins or short-chain fatty acids. Finally, controlled *in vivo* validation is performed in the target reptile species or suitable models to assess effects on growth performance, intestinal morphology, immune parameters, and resistance to experimental pathogen challenge (Figure 1).

These potential mechanisms of action are multifaceted. They include competitive exclusion of pathogens, production of antimicrobial substances such as organic acids and bacteriocins, enhancement of gut barrier integrity, and importantly-bidirectional interactions with the host immune system. Microbial-associated molecular patterns (MAMPs) interact with host pattern-recognition receptors to modulate immune responses, while the host immune system in turn shapes the composition and function of the gut microbiota (Bletz et al., 2016; Muegge et al., 2011; Siddiqui et al., 2022). This regulatory relationship, rather than a simple parallel input, underlies the potential of probiotics to promote a balanced immune state. However, significant translational challenges exist. Host specificity is a major hurdle; a probiotic strain effective in a mammalian system may fail to colonize or function in a reptile due to profound differences in core body temperature, digestive pH, gut transit time, and host immune recognition molecules. Product formulation and stability for ectothermic pets, ensuring viability during storage and passage through a variable-temperature GI tract, is non-trivial. There are also ecological risks associated with introducing non-native microbial strains, which could potentially disrupt indigenous community networks, become pathogenic under certain conditions, or facilitate unintended horizontal gene transfer of virulence or resistance factors. Despite these hurdles, the potential rewards for developing host-adapted probiotics to improve reptile health, reduce prophylactic antibiotic use, and manage specific enteric diseases are substantial and warrant continued investment (Broens and Van Geijlswijk, 2018).

### 3.2 The gut as a zoonotic pathogen reservoir

**Figure 2:**
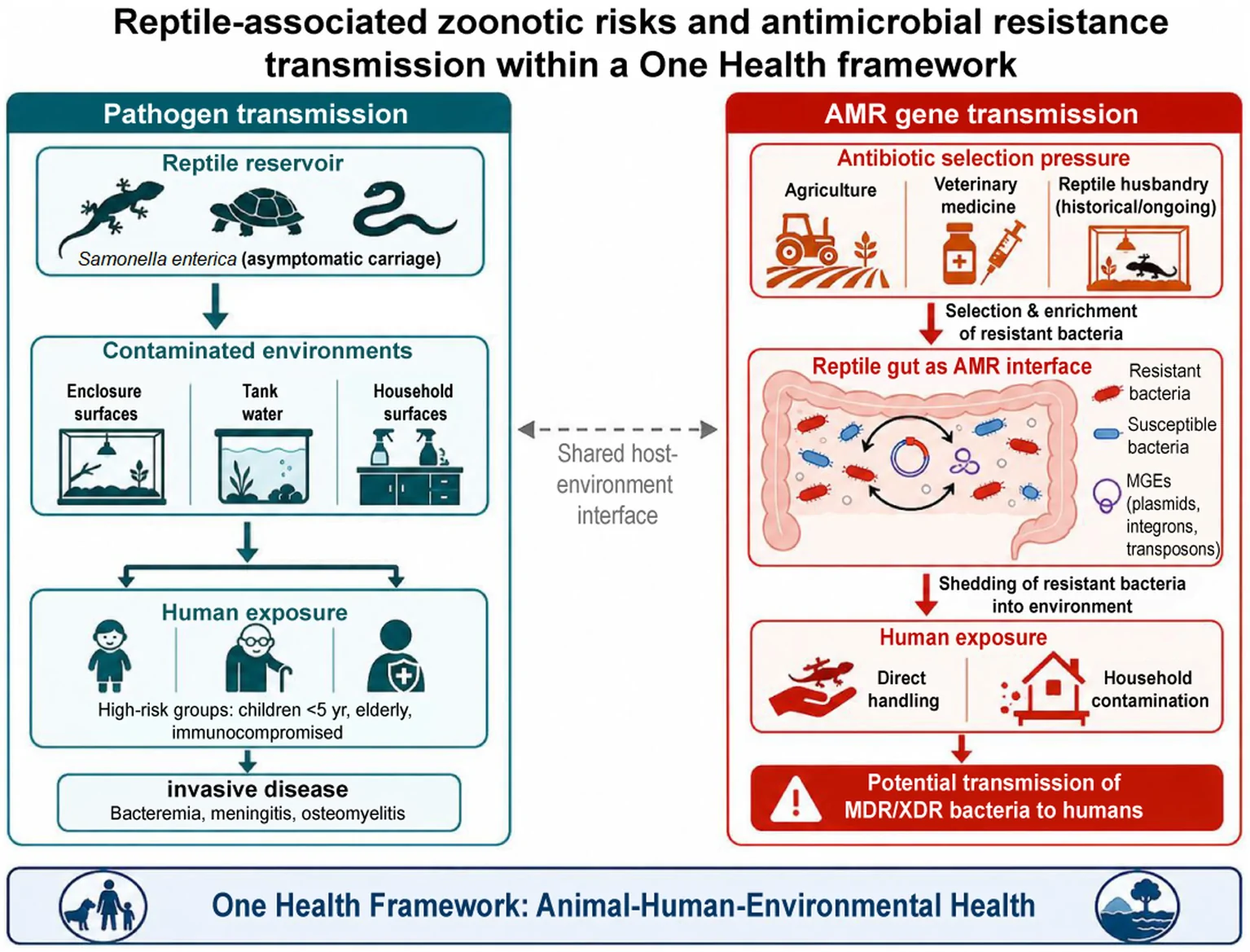
Overview of reptile-associated zoonotic risks within a One Health framework. The left panel illustrates transmission pathways of zoonotic enteric pathogens (primarily non-typhoidal *Salmonella*) from reptile reservoirs to humans via direct contact, contaminated environments, and husbandry practices. The right panel depicts antimicrobial resistance gene flow: Antibiotics used in agriculture and veterinary medicine select for resistant bacteria, which can colonize the reptilian gut; resistance genes carried on mobile genetic elements may be horizontally transferred within the gut microbial community and potentially transmitted to humans, highlighting the reptile gut as an interface in the environmental-animal-human AMR continuum.

Paradoxically, the same gut environment that may harbor beneficial microbes is also a globally recognized reservoir for zoonotic pathogens. *S. enterica* is the most prominent and well-studied, often existing as a commensal or latent resident in clinically healthy reptiles, with colonization rates varying by species, as previously noted. Its transition from commensal to pathogen is highly context-dependent, frequently triggered by host immunosuppression from concurrent viral infections like ferlaviruses in snakes, ranaviruses in chelonians, environmental stressors, poor husbandry leading to immune compromise, or concurrent parasitic burdens (Pees et al., 2023). In reptiles themselves, *Salmonella* spp. can cause severe, often systemic diseases including septicemia, granulomatous pneumonia, embolic osteomyelitis, and necrotizing enterocolitis. The serovar and subspecies epidemiology is non-random; Subspecies I (*S. enterica* subsp. *enterica*) is most commonly detected in reptile guts (~ 70% of isolates) and is overwhelmingly responsible for Reptile-Associated Salmonellosis (RAS) in humans, accounting for ~ 90% of cases (Corrente et al., 2017). Other enteric bacteria with confirmed zoonotic potential isolated from reptiles include *C. jejuni*/*C. coli*, *Aeromonas hydrophila*, *Pseudomonas aeruginosa*, and specific pathovars of *E. coli*, though their prevalence and disease significance in both reptiles and humans linked to direct reptile contact are less comprehensively documented than for *Salmonella* (Jia et al., 2025; Figure 2, left panel).

### 3.3 Interactions among host, microbiome, and parasites

The reptilian gut is also a common habitat for a variety of parasitic organisms, particularly protozoans like *Cryptosporidium* spp. and various nematodes, cestodes, and pentastomids. The relationship between parasites and the resident gut microbiota is bidirectional, dynamic, and can significantly influence disease outcome. Parasitic infection can induce significant dysbiosis, often reducing overall microbial alpha diversity and enriching for potential bacterial pathobionts or pro-inflammatory taxa through mechanisms involving direct physical damage to the mucosal lining, creation of new ecological niches in damaged tissue, alteration of nutrient availability and redox potential, and the triggering of potent host type 2 inflammatory and immune responses that dramatically change the gut luminal environment (Trevelline et al., 2019). Conversely, the resident microbiome can profoundly influence parasite establishment, fecundity, and pathogenicity. A healthy, diverse, and stable microbiota may provide colonization resistance by competing for nutrients and attachment sites, by producing metabolites directly inhibitory to the parasite, or by stimulating appropriate host immune pathways such as Th1/Th17 responses, that limit parasitic invasion and replication (Hird, 2017). Alternatively, a pre-existing dysbiotic state, characterized by a loss of protective SCFA-producing bacteria like *Clostridia* and an expansion of *Proteobacteria*, can compromise gut barrier function and lead to dysregulated immune tolerance, thereby increasing host susceptibility to parasites like *Cryptosporidium*, leading to a vicious cycle of inflammation, villous atrophy, malabsorption, and worsening microbial imbalance. Furthermore, parasite-induced dysbiosis can create conditions favorable for the expansion of antimicrobial-resistant bacterial populations. Inflammatory states alter the gut luminal environment, increasing the availability of reactive oxygen species and changing the expression of bacterial efflux pumps, both of which can promote the enrichment of resistant strains even in the absence of direct antibiotic selection (Jia et al., 2025). Concurrent parasitic infections may therefore indirectly enhance the risk of ARG dissemination by destabilizing the resident community and creating ecological opportunities for resistant opportunists. This tripartite interaction between host, microbiome, and parasite is a critical area for future research, with implications for understanding disease susceptibility and developing integrated treatment strategies (Pees et al., 2023).

### 3.4 Antimicrobial resistance in the reptilian gut

A pressing contemporary concern, positioned at the heart of the One Health crisis, is the role of the reptilian gut as a reservoir and potential amplifier for antimicrobial resistance genes (ARGs). This represents a critical and under-surveyed interface, as mobile genetic elements (MGEs) such as plasmids, integrons, and transposons carrying diverse ARGs can be efficiently horizontally transferred between commensal bacteria (*E. coli*, *Enterococcus* spp.) and zoonotic pathogens like *Salmonella* or *Campylobacter* within the densely populated, biofilm-prone gut milieu. This intra-gut transfer can create multidrug-resistant (MDR) or even extensively drug-resistant (XDR) strains that complicate clinical treatment and threaten last-resort antibiotics. Studies from North America, Europe, and Asia consistently confirm the frequent isolation of MDR Salmonella from both captive and wild reptiles (Costello et al., 2010). The drivers shaping reptile microbiomes and their environments are multifaceted. These include the historical and sometimes ongoing use of antibiotics in commercial breeding for therapy or disease prevention. They also stem from environmental contamination via human sewage and agricultural runoff, which introduces antibiotics and resistant bacteria. Furthermore, the dietary intake of commercially raised feeder animals, which may be reared with antibiotics, can introduce multi-drug resistant bacteria into the predator’s gut. Large-scale genomic surveillance projects, such as the analysis of nearly 8,000 *Salmonella* genomes from China, reveal a complex, temporally dynamic, and geographically structured landscape of AMR, with significant shifts in resistance patterns linked to changes in agricultural and veterinary antibiotic usage practices over decades (Wang et al., 2023, 2025). The reptilian intestine, with its variable temperature, potential for inflammatory states, and diverse microbial density, may provide favorable conditions for horizontal gene transfer (Kreisinger et al., 2017). The transmission chain of antimicrobial resistance involves multiple connected steps as follows: (i) antibiotic use in agriculture, veterinary medicine, and historically-reptile husbandry selects for resistant bacteria in the environment and in feeder animals; (ii) resistant bacteria and residual antibiotics enter the reptilian gut through diet, water, and environmental exposure; (iii) within the gut, mobile genetic elements such as plasmids, integrons, and transposons facilitate the transfer of resistance genes between commensals and potential pathogens; and (iv) resistant bacteria may be shed into the household or captive environment, creating potential exposure pathways to humans. This framework positions the reptile gut as a potential node in the “environmental-animal-human” AMR continuum, though direct evidence for each step remains incomplete and represents an important research priority. Recent large-scale metagenomic investigations across diverse host populations have provided compelling evidence that MGEs serve as key drivers of resistome expansion and dissemination across ecological boundaries (Tian et al., 2025a; Tian et al., 2025b; Tian and Zhang, 2025). Notably, socioeconomic gradients, particularly income level and industrialization, have been associated with increased abundance of MGEs and their associated resistance genes in human gut microbiomes, suggesting that lifestyle and environmental exposures exert strong selective pressures on the mobilome (Tian et al., 2025b; Tian and Zhang, 2025). These observations, when viewed alongside the extensive international pet trade that moves millions of reptiles across geographic and socioeconomic boundaries each year, highlight an urgent need to characterize the reptilian gut mobilome and its potential for bidirectional gene flow with human-associated microbial communities (Figure 2, right panel).

### 3.5 Metabolic contributions in fermentation, nutrient synthesis, and health

Metabolically, the gut microbiota is an indispensable partner for the host, particularly for non-carnivorous species. In herbivorous and omnivorous reptiles, microbial fermentation of dietary fiber, resistant starch, and other complex carbohydrates in the hindgut is a primary source of energy, yielding SCFAs, notably acetate, propionate, and butyrate (Song et al., 2020). Butyrate serves as the preferred energy source for colonocytes, reinforcing the gut epithelial barrier, stimulating mucin production, and exerting potent anti-inflammatory effects. Propionate is primarily metabolized in the liver for gluconeogenesis, while acetate enters systemic circulation to participate in cholesterol and fatty acid metabolism (Obregon-Tito et al., 2015). Beyond energy harvest, the microbiota plays a central role in nitrogen recycling, including the conversion of host-derived urea to microbial protein, and in synthesizing essential vitamins that the host cannot produce, such as B12, folate, biotin, and vitamin K (Gomez et al., 2016). The production profile of these metabolites directly underpins host health; a balanced, resilient microbiome with robust SCFA output supports intestinal homeostasis, anti-inflammatory signaling, and efficient energy harvest. In contrast, ecological imbalance, which refers to the transformation of beneficial fermentation bacteria into spoilage or pathogenic bacterial groups, can lead to a decrease in SCFA levels, accumulation of harmful metabolites, such as ammonia and branched chain fatty acids, intestinal barrier leakage, low-grade inflammation throughout the body, and increased susceptibility to intestinal and systemic diseases (Martínez et al., 2015).

Importantly, the shift from a balanced fermentative community to a dysbiotic state has direct implications for antimicrobial resistance. A compromised intestinal barrier, resulting from reduced butyrate production and diminished epithelial integrity, facilitates the translocation of viable bacteria, including ARG-carrying strains, across the mucosal surface (Martínez et al., 2015). Once these bacteria reach systemic sites, they may seed extraintestinal infections that are more difficult to treat due to their resistance profiles. Moreover, the accumulation of inflammatory metabolites and reduced SCFA levels can alter the local selective environment, potentially favoring the proliferation of facultative anaerobes belonging to the Proteobacteria, a phylum disproportionately enriched for clinically relevant ARGs (Kreisinger et al., 2017). Thus, metabolic dysbiosis and resistome expansion are functionally intertwined, and interventions aimed at restoring metabolic homeostasis may also serve to mitigate ARG dissemination.

## 4 Health implications, zoonotic risks and companion animal interface

### 4.1 The burden, epidemiology and transmission pathways of reptile-associated zoonoses

The public health significance of the reptilian gut microbiome is most acutely and consistently demonstrated by zoonotic transmission, primarily of non-typhoidal *Salmonella*. Reptiles, particularly popular pet species, are a major, persistent source of human salmonellosis, with epidemiological models estimating that approximately 6% of all sporadic human salmonellosis cases in some developed countries are attributable to direct or indirect contact with reptiles (Pees et al., 2023). Interestingly, epidemiological patterns often reveal a disconnect between carriage prevalence in healthy animals and outbreak association. Turtles, despite a lower reported *Salmonella* prevalence in systematic surveys, are frequently implicated in multi-state outbreaks (35.3% of RAS cases), likely due to their historical and ongoing popularity as “starter pets” for young children and their characteristic aquatic husbandry (Bosch et al., 2016). Water in turtle tanks readily becomes contaminated with feces, facilitating environmental persistence and spread, and high-risk behaviors like kissing turtles, allowing them to roam on food preparation surfaces, or cleaning enclosures in kitchen sinks are commonly reported (De Jong et al., 2005). Transmission pathways are diverse and often indirect, occurring not only through direct handling but also via contaminated enclosure surfaces, water, feeder insects or rodents, and household environments. Alarmingly, reptile-associated *Salmonella* can persist in household environments for weeks or even months after an animal’s removal, as detected on surfaces and in vacuum cleaner dust (Pees et al., 2023). Clinical outcomes are often more severe in vulnerable populations, with children under 5 years, the elderly, and immunocompromised individuals at highest risk for severe, invasive disease, including bacteremia, septicemia, meningitis, and osteomyelitis, leading to higher rates of hospitalization compared to foodborne salmonellosis from other sources (Waite and Taylor, 2015).

The companion animal reptile microbiome presents a specific, intensifying, and poorly regulated One Health challenge. The global exotic pet trade involves the movement of tens of millions of live reptiles annually, spanning species from leopard geckos (*Eublepharis macularius*) and crested geckos to ball pythons and bearded dragons (Marshall et al., 2020). These animals are often mass-produced in large-scale breeding facilities, where biosecurity, veterinary oversight, and antibiotic stewardship practices can be highly variable and sometimes suboptimal (Jiménez and Sommer, 2017). Consequently, pet reptiles may enter retail stores and ultimately private households already colonized by a gut microbiome shaped by these conditions, one that potentially includes not only zoonotic *Salmonella* but also a diverse and abundant array of ARGs on mobile genetic elements (Figure 2). Within the home, suboptimal husbandry and hygiene practices, such as cleaning enclosures in kitchen or bathroom sinks, inconsistent handwashing after handling or maintenance, and allowing reptiles to roam freely in living areas, dramatically amplify the risk of bidirectional microbial exchange (Kueneman et al., 2014). This dynamic effectively transforms the household into a micro-scale One Health unit, where the microbiome and, critically, the resistome of a pet reptile could potentially influence the local microbial ecology, posing a direct, though often unrecognized and insidious, risk of transmitting both pathogens and resistance determinants to human occupants, especially young children (Figure 2). Surveys consistently indicate a critical and pervasive knowledge gap among owners. Awareness of the salmonellosis risk is often low, and even among those with some awareness, compliance with recommended hygiene measures is frequently poor (Žahourek and Kvičerová, 2025). This highlights an urgent need for targeted, accessible, and repeated education alongside potential regulatory measures, such as mandating clear, written point-of-sale information on zoonotic risks, mirroring policies enacted for small turtles in some jurisdictions (Mermin et al., 2004).

### 4.2 The comparative and translational value for advancing human health and therapy

Beyond the direct risks, the study of reptilian gut ecosystems offers valuable comparative insights for human health and novel therapeutic discovery. From an evolutionary biology perspective, comparing the gut ecosystems of reptiles, birds, and mammals helps reconstruct the deep history of vertebrate-microbe symbioses and identify core principles of community assembly and host control that are independent of endothermy and placental reproduction. More directly, the proven capacity of some reptile-associated bacteria to produce novel antimicrobials against ESKAPE pathogens is of high biomedical interest in an era of escalating antibiotic resistance and a stagnant discovery pipeline (Akbar et al., 2019a). Furthermore, understanding the immunological and physiological mechanisms that allow many reptiles to peacefully co-exist with potent pathogens like *Salmonella*, essentially a state of “disease tolerance” rather than sterile resistance, could reveal novel immunomodulatory pathways, regulatory cell types, or metabolic adaptations that might be harnessed therapeutically to treat human inflammatory bowel diseases, sepsis, or to induce tolerance in transplant medicine (Carmody et al., 2015).

### 4.3 Modulating the microbiome for conservation and captive health management

In applied reptile conservation and captive management, strategic modulation of the gut microbiome holds transformative potential. For endangered species in captive assurance and breeding-for-reintroduction programs, interventions like fecal microbiota transplantation (FMT) from healthy, wild donor conspecifics or administration of carefully designed, multi-strain probiotic consortia could be used to “rewild” or optimize the gut microbiome of captive-born individuals (West et al., 2019). This may improve digestive efficiency and adaptation to naturalistic diets, enhance innate and adaptive immune competence, reduce the incidence and severity of enteric disease outbreaks, and increase overall phenotypic robustness and fitness. The approaches could directly boost reproductive success, juvenile survival rates, and potentially the post-release survival and ecological competence of animals destined for reintroduction, addressing a major challenge in conservation translocations (Kohl, 2020). In commercial herpetoculture, zoological institutions, and private collections, developing evidence-based, species- or diet-specific probiotic and prebiotic strategies offers a sustainable, proactive alternative to antibiotics for disease prevention and health promotion (Cullen et al., 2020). This aligns perfectly with global antimicrobial stewardship efforts and has the potential to improve animal welfare by fostering a stable, resilient, and functional gut ecosystem, reducing stress-related gastrointestinal disorders and improving nutrient utilization. A key component of this is reducing or eliminating the prophylactic use of antibiotics, especially those classified by the World Health Organization (WHO) as Highest Priority Critically Important Antimicrobials (HPCIA), in reptile production systems to curtail the amplification and spread of AMR within these populations and beyond (Leeming et al., 2021).

## 5 Methodological advances, integration and future research imperatives

### 5.1 Current technological and analytical toolkit

Elucidating the profound complexity of the reptilian gut microbiome has been propelled by rapid technological advancements in molecular biology and bioinformatics. 16S rRNA amplicon sequencing remains a cost-effective workhorse for large-scale profiling of bacterial and archaeal diversity and community structures (Zhu et al., 2025). However, to access the full functional gene repertoire, identify virulence factors, and comprehensively profile ARGs and mobile genetic elements, shotgun metagenomics is essential (Wang et al., 2025). This technique enables the reconstruction of metagenome-assembled genomes (MAGs), allowing for population genetic analyses and the assignment of functional potential to specific microbial lineages. Metatranscriptomics (RNA-seq) reveals which genes are actively being expressed under different conditions, providing a dynamic view of community function and host-microbe dialogue. Metabolomics, particularly using mass spectrometry to profile SCFAs, bile acids, and other microbial metabolites in feces or intestinal content, directly measures the functional output of the microbiome and its chemical interaction with the host (Akbar et al., 2020). Sophisticated bioinformatics pipelines are the indispensable backbone of this work.

### 5.2 Persistent challenges and knowledge gaps

Despite significant progress, the field confronts substantial challenges and knowledge gaps that hinder translation and comprehensive understanding. Many studies remain cross-sectional with limited sample sizes, lacking the longitudinal, repeated-measures data required to infer causality, understand temporal stability, and disentangle the effects of co-varying factors. The recent development of curated cross-host databases, such as the Animal Microbiome Database (AMDB), which integrates 16S rRNA gene profiles with manually curated metadata from over 400 animal species, has facilitated comparative analyses across vertebrate gut microbial communities (Yang et al., 2022). However, the representation of reptiles in such resources remains disproportionately low relative to their species richness, limiting the statistical power of reptile-focused meta-analyses and the ability to identify host-specific versus broadly conserved patterns in gut microbiome assembly. There is a significant “plate count abnormality” phenomenon in the gut of reptiles, and over 90% of microbial diversity still cannot be cultured using standard techniques (Lagier et al., 2018; Steen et al., 2019). This bottleneck seriously hinders functional validation, physiological research, and the development of probiotic products. Tools for functional annotation of metagenomic data, such as HMMER-based extractors for genomic macromolecular metabolites, have improved the ability to link metagenomic sequences to potential functions, yet their application to reptile gut datasets remains nascent (Yang et al., 2024). The precise molecular mechanisms linking specific microbial taxa, their genes, or their metabolites to reptilian host physiology, development, and behavior are largely unknown. Furthermore, standardized bioinformatic pipelines are critical for cross-study comparisons, specialized databases for screening specific metabolite-associated pathways, such as trimethylamine N-oxide (TMAO)-associated cardiovascular disease markers, exemplify the value of consistent annotation frameworks that are currently lacking for reptile microbiome research (Yang et al., 2025a).

### 5.3 Essential future research directions

Future research must prioritize several key and interrelated fields to advance the field from descriptive ecology to predictive science and applied solutions. Firstly, longitudinal and controlled experimental studies are needed to track individuals from hatching to critical life stages and to establish causal relationships between microbiome status and host phenotype through interventions such as controlled dietary transitions, pathogen challenges, or probiotic/microbiota transplantation. Secondly, multi-omics approaches, including metagenomics, metatranscriptomics, metabolomics, and culturomics, should be integrated within a unified framework, from genetic blueprint and gene expression to functional activity and chemical output, in order to construct a comprehensive mechanistic understanding. Specialized tools for KEGG ortholog extraction and functional annotation will be essential for standardizing such multi-omics analyses across reptile studies (Zhang et al., 2023). Thirdly, emerging models such as reptile gut organoids or *in vitro* simulation systems should be utilized to dissect host–microbe interaction mechanisms at the cellular and molecular levels. Large-scale, strictly controlled intervention trials must also be conducted across different species and husbandry scenarios to evaluate the safety, efficacy, and ecological impact of specific probiotic, prebiotic, synbiotic, or microbiota transplantation regimens.

In addition, an active monitoring network based on genomic epidemiology should be established to systematically track antimicrobial resistance genes in reptile-associated bacteria throughout the trade chain and in wild populations adjacent to human activities, with data integrated alongside human clinical isolates to enable comprehensive risk assessment. The application of comprehensive microbial metabolite detection toolkits tailored to microbiome datasets, such as those that systematically catalog biosynthetic gene clusters and bioactive compounds, will further strengthen the functional interpretation of such surveillance data (Tian and Zhang, 2025). Importantly, such surveillance efforts must be designed to capture not only ARG profiles but also the broader ecological and socioeconomic determinants that shape resistome dynamics. Recent evidence from mammalian and human microbiome studies has demonstrated that host ecology, geographic isolation, industrialization, and socioeconomic status collectively drive the composition and mobility of gut resistomes (Tian et al., 2025a; Tian et al., 2025b; Tian and Zhang, 2025). These findings underscore the need for comparative, multi-host surveillance frameworks that account for environmental gradients and anthropogenic pressures when assessing AMR transmission risks from wildlife and companion animal reservoirs, including reptiles (Yang et al., 2025b). Finally, and critically, future research must position resistome characterization as an integral component of all reptile microbiome studies rather than an ancillary add-on. This means that standard 16S rRNA amplicon surveys should, whenever feasible, be complemented by shotgun metagenomic sequencing or targeted ARG panel assays to capture the resistance gene repertoire of the same communities being described taxonomically. Longitudinal sampling designs should incorporate ARG tracking alongside community dynamics to establish temporal relationships between ecological shifts and resistance gene expansion or contraction. Integrating these AMR-focused dimensions into routine microbiome investigations will ensure that the field moves beyond descriptive inventories toward a predictive understanding of when and how the reptilian gut transitions from a commensal reservoir to a source of clinically relevant resistance threats.

## 6 Synthesis and future outlook

While earlier reviews have provided foundational descriptions of reptilian gut microbial diversity, the present synthesis offers a critical update that advances the field through several distinctive contributions. First, we explicitly embed antimicrobial resistance as a central organizing theme rather than a peripheral concern, systematically tracing the sources, ecological drivers, and transmission pathways of resistance genes across the reptile–human–environment interface, a dimension that previous syntheses have treated only superficially (Pees et al., 2023; Dróżdż et al., 2021). This emphasis aligns with the growing recognition that the reptilian gut resistome, though largely uncharacterized compared to livestock and human systems, may represent a significant node in the global One Health surveillance network (Wang et al., 2023, 2025). Second, we integrate findings from studies published in 2024–2025 that leverage advanced methodologies, including shotgun metagenomics, longitudinal sampling, and cross-host comparative frameworks, to move beyond taxonomic inventories toward functional and temporal understanding of these ecosystems (Colston et al., 2025; Yang et al., 2022; Cong et al., 2025; Zhu et al., 2025). These recent contributions have begun to reveal not only community composition but also the active metabolic pathways and ecological processes that govern gut assembly in reptiles. Third, we introduce and critically evaluate the concept of a ‘functional core microbiome’ in reptiles, shifting the analytical lens from taxonomically defined cores, which remain elusive given the immense phylogenetic and ecological diversity of reptiles (Amato et al., 2015; Bletz et al., 2016), to phylogenetically diverse microbial guilds that converge on host-essential functions such as fiber fermentation, vitamin synthesis, and immune modulation, a framework previously developed in mammals but only recently speculated to extend to reptiles (Muegge et al., 2011). Despite these advances, major knowledge gaps persist: wild snake populations remain severely understudied, with the majority of available data derived from captive or commercially farmed individuals (Smith et al., 2021; Colston et al., 2025); over 90% of reptilian gut microbial diversity remains unculturable, severely constraining functional validation and probiotic development (Lagier et al., 2018; Steen et al., 2019); and the reptilian gut resistome lacks comprehensive genomic surveillance across the pet trade chain, limiting our ability to assess transmission risks to human populations (Corrente et al., 2017; Wang et al., 2025). By explicitly articulating these breakthroughs alongside persistent blind spots, this review aims to chart a forward-looking research agenda that transitions from descriptive surveys toward mechanistic interrogation, predictive modeling, and evidence-based translational applications in captive management, conservation breeding, and zoonotic risk mitigation.

The gut microbiome of reptiles is a complex and dynamic ecosystem situated at the intersection of biology, medicine, and environmental science. Far more than a digestive aid, it is a co-evolutionary cornerstone of reptile physiology, shaping nutritional biology, immune development, and adaptability. This microbial community represents both a reservoir of genetic diversity with untapped therapeutic potential and a persistent source of zoonotic pathogens and antimicrobial resistance genes. This inherent duality, of being both an opportunity and a risk, is what fundamentally defines and drives the current research landscape. Addressing it requires sustained interdisciplinary commitment: closing knowledge gaps in host–microbe interactions, developing evidence-based policies across the reptile trade, and fostering communication between microbiologists, veterinarians, regulators, and the public.

Deepened exploration of the reptilian gut, guided by ecological and evolutionary principles, promises insights that extend far beyond herpetology. It will inform comparative physiology, support biodiversity conservation, and offer novel solutions to the antimicrobial resistance crisis. Ultimately, understanding this hidden microbial world is essential not merely as an academic pursuit, but as a practical imperative for safeguarding health across an interconnected biosphere. The integration of gut microbial ecology with antimicrobial resistance surveillance, from the captive breeding facility to the household terrarium, represents a frontier that sits at the intersection of basic biology, clinical medicine, and environmental health. Advancing this integration will require not only technological innovation and interdisciplinary collaboration but also policy frameworks that recognize the reptile gut as a legitimate node in the global One Health network.

## Footnotes

1 AMDB, http://leb.snu.ac.kr/amdb/.
