# Supplementary material for: Microbial diversity, antimicrobial resistance and zoonotic implications of the reptile gut microbiota: an updated review
Source: Front Microbiol. 2026 Sep 1;17:1913228. doi: 10.3389/fmicb.2026.1913228 (PMC13575676; doi:10.3389/fmicb.2026.1913228)
Supplement: Supplementary file 1 [file Data_Sheet_1.PDF]

Table S1. List of individual 16S rRNA gene accessions from reptile gut datasets.

**Accession**	**Project**	**Host**	**Host taxonomy**	**Host diet**	**Captivity**
ERS2859665	PRJEB29403	Aesculapian snake	Chordata; Reptilia; Squamata; Colubridae; Zamenis; Zamenis longissimus	Carnivore	Wild
SRS1930148	PRJNA362214	Aldabra giant tortoise	Chordata; Reptilia; Testudines; Testudinidae; Aldabrachelys; Aldabrachelys gigantea	Herbivore	Captive
SRS1930149	PRJNA362214	Aldabra giant tortoise	Chordata; Reptilia; Testudines; Testudinidae; Aldabrachelys;	Herbivore	Captive
ERS4037916	PRJEB35449	Aldabra giant tortoise	Chordata; Reptilia; Testudines; Testudinidae; Aldabrachelys;	Herbivore	Captive
ERS4037919	PRJEB35449	Aldabra giant tortoise	Chordata; Reptilia; Testudines; Testudinidae; Aldabrachelys;	Herbivore	Captive
ERS4038703	PRJEB35449	Aldabra giant tortoise	Chordata; Reptilia; Testudines; Testudinidae; Aldabrachelys;	Herbivore	Captive
ERS4038106	PRJEB35449	Allisons anole	Chordata; Reptilia; Squamata; Iguanidae; Anolis; Anolis allisoni	Carnivore	Captive
ERS4038128	PRJEB35449	Allisons anole	Chordata; Reptilia; Squamata; Iguanidae; Anolis; Anolis allisoni	Carnivore	Captive
ERS4037946	PRJEB35449	Amazon tree boa	Chordata; Reptilia; Squamata; Boidae; Corallus; Corallus hortulana	Carnivore	Captive
ERS4038705	PRJEB35449	American alligator	Chordata; Reptilia; Crocodilia; Alligatoridae; Alligator; Alligator	Carnivore	Captive

ERS4038009	PRJEB35449	Ball python	Chordata; Reptilia; Squamata; Pythonidae; Python; Python regius	Carnivore	Captive
ERS4037939	PRJEB35449	Bearded dragon	Chordata; Reptilia; Squamata; Agamidae; Pogona; Pogona vitticeps	Omnivore	Captive
ERS4037940	PRJEB35449	Bearded dragon	Chordata; Reptilia; Squamata; Agamidae; Pogona; Pogona vitticeps	Omnivore	Captive
ERS4037943	PRJEB35449	Bearded dragon	Chordata; Reptilia; Squamata; Agamidae; Pogona; Pogona vitticeps	Omnivore	Captive
ERS4037944	PRJEB35449	Bearded dragon	Chordata; Reptilia; Squamata; Agamidae; Pogona; Pogona vitticeps	Omnivore	Captive
ERS4037948	PRJEB35449	Bearded dragon	Chordata; Reptilia; Squamata; Agamidae; Pogona; Pogona vitticeps	Omnivore	Captive
ERS4037949	PRJEB35449	Bearded dragon	Chordata; Reptilia; Squamata; Agamidae; Pogona; Pogona vitticeps	Omnivore	Captive
ERS4037962	PRJEB35449	Bearded dragon	Chordata; Reptilia; Squamata; Agamidae; Pogona; Pogona vitticeps	Omnivore	Captive
ERS4038120	PRJEB35449	Bearded dragon	Chordata; Reptilia; Squamata; Agamidae; Pogona; Pogona vitticeps	Omnivore	Captive
ERS4037895	PRJEB35449	Black throated monitor	Chordata; Reptilia; Squamata; Varanidae; Varanus; Varanus albigularis	Carnivore	Captive
ERS4038399	PRJEB35449	Boa constrictor	Chordata; Reptilia; Squamata; Boidae; Boa; Boa constrictor	Carnivore	Captive

ERS4038401	PRJEB35449	Boa constrictor	Chordata; Reptilia; Squamata; Boidae; Boa; Boa constrictor	Carnivore	Captive
ERS4038669	PRJEB35449	Burmese python	Chordata; Reptilia; Squamata; Pythonidae; Python; Python bivittatus	Carnivore	Captive
ERS4037987	PRJEB35449	Burmese star tortoise	Chordata; Reptilia; Testudines; Testudinidae; Geochelone;	Herbivore	Captive
ERS4037989	PRJEB35449	Burmese star tortoise	Chordata; Reptilia; Testudines; Testudinidae; Geochelone;	Herbivore	Captive
ERS4038569	PRJEB35449	Caiman lizard	Chordata; Reptilia; Squamata; Teiidae; Dracaena; Dracaena guianensis	Omnivore	Captive
ERS4038684	PRJEB35449	Chameleon forest dragon	Chordata; Reptilia; Squamata; Agamidae; Gonocephalus; Gonocephalus	Carnivore	Captive
ERS4038627	PRJEB35449	Chameleon	Chordata; Reptilia; Squamata; Chamaeleonidae; Chamaeleo; Chamaeleo	Omnivore	Captive
SRS4297936	PRJNA516815	Chinese cobra	Chordata; Reptilia; Squamata; Elapidae; Naja; Naja atra	Carnivore	Captive
SRS4297935	PRJNA516815	Chinese cobra	Chordata; Reptilia; Squamata; Elapidae; Naja; Naja atra	Carnivore	Captive
SRS4297932	PRJNA516815	Chinese cobra	Chordata; Reptilia; Squamata; Elapidae; Naja; Naja atra	Carnivore	Captive
SRS4297931	PRJNA516815	Chinese cobra	Chordata; Reptilia; Squamata; Elapidae; Naja; Naja atra	Carnivore	Captive

SRS4297930	PRJNA516815	Chinese cobra	Chordata; Reptilia; Squamata; Elapidae; Naja; Naja atra	Carnivore	Captive
SRS4297929	PRJNA516815	Chinese cobra	Chordata; Reptilia; Squamata; Elapidae; Naja; Naja atra	Carnivore	Captive
ERS4038691	PRJEB35449	Chinese crocodile lizard	Chordata; Reptilia; Squamata; Shinisauridae; Shinisaurus;	Carnivore	Captive
ERS4038688	PRJEB35449	Chinese crocodile lizard	Chordata; Reptilia; Squamata; Shinisauridae; Shinisaurus;	Carnivore	Captive
SRS4297926	PRJNA516815	Chinese moccasin	Chordata; Reptilia; Squamata; Viperidae; Deinagkistrodon; Deinagkistrodon acutus	Carnivore	Captive
SRS4297925	PRJNA516815	Chinese moccasin	Chordata; Reptilia; Squamata; Viperidae; Deinagkistrodon; Deinagkistrodon acutus	Carnivore	Captive
SRS4297924	PRJNA516815	Chinese moccasin	Chordata; Reptilia; Squamata; Viperidae; Deinagkistrodon; Deinagkistrodon acutus	Carnivore	Captive
SRS4297923	PRJNA516815	Chinese moccasin	Chordata; Reptilia; Squamata; Viperidae; Deinagkistrodon; Deinagkistrodon acutus	Carnivore	Captive
SRS4297922	PRJNA516815	Chinese moccasin	Chordata; Reptilia; Squamata; Viperidae; Deinagkistrodon; Deinagkistrodon acutus	Carnivore	Captive
SRS4297921	PRJNA516815	Chinese moccasin	Chordata; Reptilia; Squamata; Viperidae; Deinagkistrodon; Deinagkistrodon acutus	Carnivore	Captive
ERS4038643	PRJEB35449	Chinese water dragon	Chordata; Reptilia; Squamata; Agamidae; Physignathus; Physignathus	Carnivore	Captive

ERS2859654	PRJEB29403	Common house gecko	Chordata; Reptilia; Squamata; Gekkonidae; Hemidactylus; Hemidactylus frenatus	Carnivore	Wild
ERS4038634	PRJEB35449	Cuban crocodile	Chordata; Reptilia; Crocodilia; Crocodylidae; Crocodylus; Crocodylus	Carnivore	Captive
ERS4038702	PRJEB35449	Cuban crocodile	Chordata; Reptilia; Crocodilia; Crocodylidae; Crocodylus; Crocodylus	Carnivore	Captive
ERS4038723	PRJEB35449	Cuban crocodile	Chordata; Reptilia; Crocodilia; Crocodylidae; Crocodylus; Crocodylus	Carnivore	Captive
ERS4038740	PRJEB35449	Cuban crocodile	Chordata; Reptilia; Crocodilia; Crocodylidae; Crocodylus; Crocodylus	Carnivore	Captive
ERS4038097	PRJEB35449	Cuban false chameleon	Chordata; Reptilia; Squamata; Iguanidae; Anolis; Anolis barbatus	Carnivore	Captive
ERS4038127	PRJEB35449	Cuban false chameleon	Chordata; Reptilia; Squamata; Iguanidae; Anolis; Anolis barbatus	Carnivore	Captive
ERS4038673	PRJEB35449	Eastern indigo snake	Chordata; Reptilia; Squamata; Colubridae; Drymarchon; Drymarchon couperi	Carnivore	Captive
ERS4038044	PRJEB35449	Egyptian spiny tailed lizard	Chordata; Reptilia; Squamata; Agamidae; Uromastyx; Uromastyx aegyptia	Herbivore	Captive
ERS4038700	PRJEB35449	Giant leaf-tailed gecko	Chordata; Reptilia; Squamata; Gekkonidae; Uroplatus; Uroplatus fimbriatus	Carnivore	Captive
ERS4037930	PRJEB35449	Glass lizard	Chordata; Reptilia; Squamata; Anguidae; Pseudopus; Pseudopus apodus	Carnivore	Captive

ERS4038698	PRJEB35449	Glass lizard	Chordata; Reptilia; Squamata; Anguidae; Pseudopus; Pseudopus apodus	Carnivore	Captive
ERS4037934	PRJEB35449	Gopher tortoise	Chordata; Reptilia; Testudines; Testudinidae; Gopherus; Gopherus	Herbivore	Captive
ERS2859595	PRJEB29403	Grass snake	Chordata; Reptilia; Squamata; Colubridae; Natrix; Natrix natrix	Carnivore	Wild
ERS4038617	PRJEB35449	Graybanded king snake	Chordata; Reptilia; Squamata; Colubridae; Lampropeltis; Lampropeltis alterna	Carnivore	Captive
ERS4038699	PRJEB35449	Green anaconda	Chordata; Reptilia; Squamata; Boidae; Eunectes; Eunectes murinus	Carnivore	Captive
ERS4038093	PRJEB35449	Green basilisk	Chordata; Reptilia; Squamata; Iguanidae; Basiliscus; Basiliscus plumifrons	Omnivore	Captive
ERS4038100	PRJEB35449	Green basilisk	Chordata; Reptilia; Squamata; Iguanidae; Basiliscus; Basiliscus plumifrons	Omnivore	Captive
ERS4038108	PRJEB35449	Green basilisk	Chordata; Reptilia; Squamata; Iguanidae; Basiliscus; Basiliscus plumifrons	Omnivore	Captive
ERS4038115	PRJEB35449	Green basilisk	Chordata; Reptilia; Squamata; Iguanidae; Basiliscus; Basiliscus plumifrons	Omnivore	Captive
ERS4038129	PRJEB35449	Green basilisk	Chordata; Reptilia; Squamata; Iguanidae; Basiliscus; Basiliscus plumifrons	Omnivore	Captive
ERS4037965	PRJEB35449	Green tree python	Chordata; Reptilia; Squamata; Pythonidae; Morelia; Morelia viridis	Carnivore	Captive

ERS4037973	PRJEB35449	Guatemalan beaded lizard	Chordata; Reptilia; Squamata; Helodermatidae; Heloderma; Heloderma	Carnivore	Captive
ERS4037988	PRJEB35449	Guatemalan beaded lizard	Chordata; Reptilia; Squamata; Helodermatidae; Heloderma; Heloderma	Carnivore	Captive
ERS4038131	PRJEB35449	Guatemalan beaded lizard	Chordata; Reptilia; Squamata; Helodermatidae; Heloderma; Heloderma	Carnivore	Captive
ERS2859598	PRJEB29403	Hermann's tortoise	Chordata; Reptilia; Testudines; Testudinidae; Testudo; Testudo hermanni	Omnivore	Wild
ERS4037968	PRJEB35449	Impressed tortoise	Chordata; Reptilia; Testudines; Testudinidae; Manouria; Manouria	Herbivore	Captive
ERS4038730	PRJEB35449	Impressed tortoise	Chordata; Reptilia; Testudines; Testudinidae; Manouria; Manouria	Herbivore	Captive
ERS4038040	PRJEB35449	Indigo snake	Chordata; Reptilia; Squamata; Colubridae; Drymarchon; Drymarchon corais	Carnivore	Captive
ERS4038629	PRJEB35449	Indochinese box turtle	Chordata; Reptilia; Testudines; Geoemydidae; Cuora; Cuora bourreti	Omnivore	Captive
ERS4037894	PRJEB35449	Iranian eyelid gecko	Chordata; Reptilia; Squamata; Eublepharidae; Eublepharis;	Carnivore	Captive
ERS4038116	PRJEB35449	Iranian eyelid gecko	Chordata; Reptilia; Squamata; Eublepharidae; Eublepharis;	Carnivore	Captive
ERS4038126	PRJEB35449	Iranian eyelid gecko	Chordata; Reptilia; Squamata; Eublepharidae; Eublepharis;	Carnivore	Captive

ERS4038130	PRJEB35449	Iranian eyelid gecko	Chordata; Reptilia; Squamata; Eublepharidae; Eublepharis;	Carnivore	Captive
ERS4038630	PRJEB35449	Iranian eyelid gecko	Chordata; Reptilia; Squamata; Eublepharidae; Eublepharis;	Carnivore	Captive
ERS2859658	PRJEB29403	Italian wall lizard	Chordata; Reptilia; Squamata; Lacertidae; Podarcis; Podarcis siculus	Carnivore	Wild
ERS4037950	PRJEB35449	Jamaican iguana	Chordata; Reptilia; Squamata; Iguanidae; Cyclura; Cyclura collei	Herbivore	Captive
SRS4297941	PRJNA516815	King ratsnake	Chordata; Reptilia; Squamata; Colubridae; Elaphe; Elaphe carinata	Carnivore	Captive
SRS4297940	PRJNA516815	King ratsnake	Chordata; Reptilia; Squamata; Colubridae; Elaphe; Elaphe carinata	Carnivore	Captive
SRS4297939	PRJNA516815	King ratsnake	Chordata; Reptilia; Squamata; Colubridae; Elaphe; Elaphe carinata	Carnivore	Captive
SRS4297934	PRJNA516815	King ratsnake	Chordata; Reptilia; Squamata; Colubridae; Elaphe; Elaphe carinata	Carnivore	Captive
SRS4297933	PRJNA516815	King ratsnake	Chordata; Reptilia; Squamata; Colubridae; Elaphe; Elaphe carinata	Carnivore	Captive
SRS4297920	PRJNA516815	King ratsnake	Chordata; Reptilia; Squamata; Colubridae; Elaphe; Elaphe carinata	Carnivore	Captive
ERS4038736	PRJEB35449	King snake	Chordata; Reptilia; Squamata; Colubridae; Lampropeltis; Lampropeltis getula	Carnivore	Captive

ERS4038050	PRJEB35449	Leopard tortoise	Chordata; Reptilia; Testudines; Testudinidae; Stigmochelys;	Herbivore	Captive
ERS4038681	PRJEB35449	Lined flat-tail gecko	Chordata; Reptilia; Squamata; Gekkonidae; Uroplatus; Uroplatus lineatus	Carnivore	Captive
ERS4038732	PRJEB35449	Lined flat-tail gecko	Chordata; Reptilia; Squamata; Gekkonidae; Uroplatus; Uroplatus lineatus	Carnivore	Captive
ERS2859573	PRJEB29403	Meadow viper	Chordata; Reptilia; Squamata; Viperidae; Vipera; Vipera ursinii	Carnivore	Wild
ERS2859697	PRJEB29403	Meadow viper	Chordata; Reptilia; Squamata; Viperidae; Vipera; Vipera ursinii	Carnivore	Wild
ERS4037951	PRJEB35449	Meller's chameleon	Chordata; Reptilia; Squamata; Chamaeleonidae; Trioceros; Trioceros	Carnivore	Captive
ERS4037963	PRJEB35449	Meller's chameleon	Chordata; Reptilia; Squamata; Chamaeleonidae; Trioceros; Trioceros	Carnivore	Captive
ERS4037967	PRJEB35449	Meller's chameleon	Chordata; Reptilia; Squamata; Chamaeleonidae; Trioceros; Trioceros	Carnivore	Captive
ERS4038030	PRJEB35449	Meller's chameleon	Chordata; Reptilia; Squamata; Chamaeleonidae; Trioceros; Trioceros	Carnivore	Captive
ERS4038094	PRJEB35449	Meller's chameleon	Chordata; Reptilia; Squamata; Chamaeleonidae; Trioceros; Trioceros	Carnivore	Captive
ERS4038098	PRJEB35449	Meller's chameleon	Chordata; Reptilia; Squamata; Chamaeleonidae; Trioceros; Trioceros	Carnivore	Captive

ERS4038101	PRJEB35449	Meller's chameleon	Chordata; Reptilia; Squamata; Chamaeleonidae; Trioceros; Trioceros	Carnivore	Captive
ERS2859699	PRJEB29403	Morelet's crocodile	Chordata; Reptilia; Crocodilia; Crocodylidae; Crocodylus; Crocodylus	Carnivore	Captive
ERS4038721	PRJEB35449	New caledonian giant gecko	Chordata; Reptilia; Squamata; Diplodactylidae; Rhacodactylus;	Omnivore	Captive
ERS2859651	PRJEB29403	Nile crocodile	Chordata; Reptilia; Crocodilia; Crocodylidae; Crocodylus; Crocodylus	Carnivore	Wild
ERS4037942	PRJEB35449	Northern watersnake	Chordata; Reptilia; Squamata; Colubridae; Nerodia; Nerodia sipedon	Carnivore	Captive
SRS4297938	PRJNA516815	Oriental ratsnake	Chordata; Reptilia; Squamata; Colubridae; Ptyas; Ptyas mucosa	Carnivore	Captive
SRS4297937	PRJNA516815	Oriental ratsnake	Chordata; Reptilia; Squamata; Colubridae; Ptyas; Ptyas mucosa	Carnivore	Captive
SRS4297928	PRJNA516815	Oriental ratsnake	Chordata; Reptilia; Squamata; Colubridae; Ptyas; Ptyas mucosa	Carnivore	Captive
SRS4297927	PRJNA516815	Oriental ratsnake	Chordata; Reptilia; Squamata; Colubridae; Ptyas; Ptyas mucosa	Carnivore	Captive
ERS4037964	PRJEB35449	Oriente knight anole	Chordata; Reptilia; Squamata; Iguanidae; Anolis; Anolis smallwoodi	Omnivore	Captive
ERS4037984	PRJEB35449	Pancake tortoise	Chordata; Reptilia; Testudines; Testudinidae; Malacochersus;	Herbivore	Captive

ERS4037992	PRJEB35449	Papuan olive python	Chordata; Reptilia; Squamata; Elapidae; Pseudechis; Pseudechis papuanus	Carnivore	Captive
ERS4037974	PRJEB35449	Ploughshare tortoise	Chordata; Reptilia; Testudines; Testudinidae; Astrochelys;	Herbivore	Captive
ERS4037996	PRJEB35449	Prehensile tailed skink	Chordata; Reptilia; Squamata; Scincidae; Corucia; Corucia zebrata	Herbivore	Captive
ERS4038704	PRJEB35449	Prehensile tailed skink	Chordata; Reptilia; Squamata; Scincidae; Corucia; Corucia zebrata	Herbivore	Captive
ERS4038565	PRJEB35449	Radiated tortoise	Chordata; Reptilia; Testudines; Testudinidae; Astrochelys;	Herbivore	Captive
ERS4038690	PRJEB35449	Radiated tortoise	Chordata; Reptilia; Testudines; Testudinidae; Astrochelys;	Herbivore	Captive
ERS4037972	PRJEB35449	Rough knob tailed gecko	Chordata; Reptilia; Squamata; Carphodactylidae; Nephrurus; Nephrurus	Carnivore	Captive
ERS4038117	PRJEB35449	Rough knob tailed gecko	Chordata; Reptilia; Squamata; Carphodactylidae; Nephrurus; Nephrurus	Carnivore	Captive
ERS4047068	PRJEB35449	Russian rat snake	Chordata; Reptilia; Squamata; Colubridae; Elaphe; Elaphe schrenckii	Carnivore	Captive
ERS4038693	PRJEB35449	Spider tortoise	Chordata; Reptilia; Testudines; Testudinidae; Pyxis; Pyxis arachnoides	Omnivore	Captive
ERS4038007	PRJEB35449	Woma python	Chordata; Reptilia; Squamata; Pythonidae; Aspidites; Aspidites ramsayi	Carnivore	Captive
